# What Do Breastfeeding Women Taking Herbal Galactagogues Perceive of Community Pharmacists’ Role in Breastfeeding Support? A Qualitative Study

**DOI:** 10.3390/ijerph120911132

**Published:** 2015-09-08

**Authors:** Tin Fei Sim, H. Laetitia Hattingh, Jillian Sherriff, Lisa B.G. Tee

**Affiliations:** 1 School of Pharmacy, Curtin University, Perth, WA 6845, Australia; E-Mails: l.hattingh@curtin.edu.au (H.L.H.); l.tee@curtin.edu.au (L.B.G.T.); 2 School of Public Health, Curtin University, Perth, WA 6845, Australia; E-Mail: j.sherriff@curtin.edu.au (J.S.)

**Keywords:** breastfeeding, infant health, community pharmacy, pharmacists, herbal medicines, women’s perspectives

## Abstract

Information from pharmacists may affect breastfeeding womens’ decisions and choice of therapy. Community pharmacies remain one of the main sources of herbal medicines in Australia. In this study, we aimed to explore the perspectives of breastfeeding women on pharmacists’ role and whether there is potential for role expansion, as well as the facilitators and barriers in meeting their healthcare-related needs in the community pharmacy setting. Semi-structured in-depth interviews were conducted with 20 Western Australian women who were using one or more herbal galactagogues while breastfeeding. Participants’ views were classified into three major themes: (i) facilitators and (ii) barriers to an increased role of pharmacists; and (iii) implementation of breastfeeding related-services in community pharmacy settings. Overall perspectives of participants were positive about the potential for role expansion of pharmacists to meet their breastfeeding-related needs. Whilst most participants perceived community pharmacies as convenient sources of trusted information, some recognised barriers to an increased role of pharmacists. Several breastfeeding support services perceived to be useful in community pharmacy settings were identified. Issues raised highlighted areas of pharmacy practice which required improvement and revealed opportunities for expansion of pharmacists’ role to better support women and promote breastfeeding in the community.

## 1. Introduction

Breastfeeding has been shown to provide healthy nutrition and contribute to the improvement of well-being of infants and mothers, leading to positive health outcomes [1]. Besides offering tailored nourishment to infants, breastfeeding also plays a role in reducing the risk and incidence of many diseases including otitis media, diabetes mellitus, hypersensitivity reactions and Sudden Infant Death Syndrome [1,2,3]. In addition to the physiological health advantages, breastfeeding may also contribute to some psychological benefits through facilitating relationships between mothers and their breastfed infants [4]. Based on many studies published over the years, breast milk is widely recommended as the exclusive feeding choice for the first six months of life, assuming it is available [5,6,7,8]. In Australia, initiation rates are high but around a third of women completely cease breastfeeding by the time their infants are four months old [9], reflecting the need for more support from health professionals. The Australian Infant Feeding Guidelines are directed at health professionals with the expressed aim of more consistent information provision to breastfeeding mothers [7].

It is estimated that 25%–35% of mothers curtail breastfeeding due to the perception that their milk supply is inadequate for their baby’s needs [7]. However, this perception can also be the cue to use some form of galactagogue. Our recent Western Australian study found that 25% of 304 survey respondents had used herbal galactagogues during breastfeeding [10]. Studies using animal models have led to possible mechanisms for their putative galactogenic effect, for example via enhanced aquaporin expression [11], but evidence for their ability to increase milk production in women rests on poor quality studies and thus their use should not be recommended [12]. It is possible that there may be psychological effects which improve self-efficacy [13,14,15] and result in continued breastfeeding. Nonetheless, this potential advantage must be balanced with any likely negative effects.

Pharmacists are included in the Australian National Breastfeeding Strategy 2010–2015 as one of the breastfeeding support staff [8]. Information from pharmacists may affect women’s experience, decisions and choice of therapy whilst breastfeeding. In addition, community pharmacies represent one of the major providers of complementary medicines (CMs) including herbal galactagogues in Australia [16]. Pharmacists are in a unique position to influence use of herbal medicines by the public as evidenced by positive consumer satisfaction rates and expectations from both Australian as well as international studies [17,18,19].

A recent Australian study demonstrated the high expectations of the general public towards community pharmacists’ knowledge and ability to provide information related to CM products including herbal medicines [19]. Several studies have also reported the perspectives and attitudes of health professionals as well as breastfeeding women towards the use of medications at this time, albeit focusing on conventional medications [20,21,22]. Nevertheless, there has been limited research to explore the perspectives of breastfeeding women towards the role of community pharmacists in relation to the use of herbal medicines and galactagogues. This study aimed to explore experiences and perspectives of breastfeeding women who use or used these products on community pharmacists’ role in this decision, as well as the facilitators and barriers in meeting their breastfeeding needs in the community pharmacy setting.

## 2. Materials and Methods 

This exploratory research was conducted through in-depth semi-structured interviews with women who were using one or more herbal galactagogues during breastfeeding. This study was approved by the Curtin University Human Research Ethics Committee (approval number HR85/2012). All participants were provided with a participant information sheet with details of the study and written consent form. Participants were informed that participation in the study was completely voluntary and that they could withdraw at any time from the study without prejudice.

### 2.1. Recruitment

Purposeful sampling was used to recruit participants, targeting breastfeeding women who had experience in using herbal medicines to enhance milk supply. The inclusion criteria were women aged 18 years or older who had been breastfeeding in the previous 12 months at the time of interview, and had used one or more herbal medicines during breastfeeding. According to Patton [23], the purposeful sampling method is powerful as it involves selection of information-rich cases which allows in-depth inquiry into the topic. Hence, careful selection of participants with experience using herbal medicines to enhance milk supply was considered appropriate to enable relevant and quality data to be collected, improving credibility of the findings.

Four different approaches were employed to recruit participants. Firstly, recruitment was targeted through clinics and a health centre. The study was promoted at three naturopathic clinics with a focus on breastfeeding and CM use. Posters with details of the study were also displayed at a health centre where agreement to collaborate in the recruitment process had been made. This health centre was located in Perth, Western Australia, where the clinic staff supported the regulation of Western herbal medicines through the Therapeutic Goods Administration (TGA) to ensure that products comply with specific quality and safety criteria.

Secondly, the study was promoted to the wider public through a media release used to promote the study in local health and parenting papers and was also announced via the Curtin FM 100.1 Perth radio station. Contact details of the primary investigator were provided to women who wanted to participate in the study.

Thirdly, a snowball sampling technique was adopted [23] and interested participants were provided with additional study information and requested to share this with other breastfeeding women. Unlike other studies where a specific participant type may be identified or recruited from an organisation or setting, the use or purchase of herbal medicines in Australia is not required to be reported nor recorded and they can be sold from many retail outlets, hence there was not a specific setting or location to recruit this specific target population. Therefore, the snowballing sampling technique fitted the scope of this study [23,24].

Fourthly, through the snowballing effect, participants suggested community pharmacies where more participants were likely to be recruited and two community pharmacies were subsequently identified for further recruitment. Both of these community pharmacies had a focus on naturopathy and CMs and provided a range of breastfeeding-related services. Participants were then recruited from these pharmacies through expression of interest in response to study posters and participant information sheets. The recruitment of participants ceased when the point of saturation was considered achieved [24,25].

### 2.2. Design and Conduct of Interviews

Interviews were conducted on a one-to-one basis, either face-to-face at a place convenient to each participant or via telephone if preferred by the participant. An interview guide was developed with a mix of closed (10) and open-ended (7) questions to gather information about the use of herbal medicines during breastfeeding, and explore the perspectives of breastfeeding women towards the role of pharmacists in meeting their breastfeeding-related healthcare needs in the community pharmacy setting. The interview guide was developed based on an initial literature review which identified a need for more research in the area of herbal galactagogue use during breastfeeding and the role of pharmacists in supporting breastfeeding women’s needs in the Australian healthcare system. The first step involved in developing the interview guide emanated from the aims and objectives of the study, and to identify exactly what information was needed to answer the research objectives. The research team then developed the interview questions required to explore issues and obtain the relevant information from participants. The interview guide was piloted on two breastfeeding women. Considering the variability between participants, the interviewer (TFS) adhered to a sensitive and flexible approach throughout the interview process to ensure the topic of discussion could be thoroughly covered. As participants were mothers with a young child, all of them were reassured that priority would be given to their infant or child if they needed to be attended to, and interviews were paused and resumed when convenient for the participant. Qualitative narrative data were collected through recordings of the dialogues between the researcher and each individual participant.

### 2.3. Data Analysis

All interviews were audio-recorded and manually transcribed verbatim. Participants were de-identified and codes used in the analysis. For example: the first interviewee was given a code “BW1”. The seven open-ended questions in the interview guide which explored the experiences and perspectives of participants were analysed using thematic analysis [26]. Firstly, contents of the transcriptions were read repeatedly by the primary investigator to attain a thorough understanding of topics that emerged from the interviews, at the same time paying attention to the “patterns” that occurred throughout the interviews. After familiarisation, the primary investigator then carefully read the transcripts line-by-line, while highlighting phrases or sentences and generating initial “codes”. Notes were made alongside the codes to describe the meaning of the codes where relevant. These codes were then grouped into categories, to form a working analytical framework. The codes were reviewed and emerged “ideas” or themes were recorded and supporting quotations documented under each theme category. Different “units of ideas” were then reclassified as subthemes under a specific “collective idea” or theme. These themes were then regrouped under distinctive headings addressing the research questions. To ensure reliability of the process of analysis, project supervisors (LH, LT) reviewed the themes and provided input throughout the data analysis process.

## 3. Results 

A total of 20 in-depth semi-structured interviews were undertaken with breastfeeding women living in the Perth metropolitan area between October 2012 and April 2013. Saturation of data was approaching after approximately 15 interviews but a decision was made to continue until all 20 participants were interviewed, after which the research team was confident that no new themes were emerging. Ten interviews were conducted face-to-face and ten via telephone. Out of the 20 participants, 18 were of Caucasian descent and two of Asian descent. Interviews took an average of 33.9 min (range: 18–78 min).

Although participants’ views varied widely, they perceived community pharmacy in general as a convenient source of information which can be trusted. When asked whether they believed that there was a role for community pharmacists to play in the area of herbal medicines and breastfeeding, common facilitating factors and barriers were identified. Throughout the interviews, participants identified several breastfeeding support services perceived to be useful and beneficial in the community pharmacy setting. Three major themes emerged as the participants described their perspectives, summarised in Table 1.

**Table 1:** Major themes and subthemes.

Main Themes	Subthemes
1. Facilitators	Convenience and accessibility
	Client-pharmacist relationship
	Staff knowledge and credibility
	Cost factors
2. Barriers	Lack of publicity and public awareness
	Inconsistent approach
	Breastfeeding-related inexperience and low awareness
	Pharmacists’ pre-conceived perception towards herbal medicines
	Overlap of role with other health professionals
	Privacy issues and pharmacy layout
3. Breastfeeding-related services	Baby weigh-in service or station
	Lactation booth
	Distribution of pamphlets/educational materials
	Information sessions in-store
	One-on-one counselling service in consultation room

### 3.1. Facilitators to an Increased Role of Community Pharmacists in Supporting Breastfeeding 

Participants identified several facilitating factors which supported the increased role of community pharmacists, including convenience and accessibility, client-pharmacist relationship, staff knowledge and credibility, and cost factors. These facilitators are summarised in Table 2 and explored below. Participant quotes are included to illustrate concepts.

**Table 2:** Facilitators to an increased role of community pharmacists.

Subthemes	Supporting Quotes
Convenience and accessibility	*“I didn’t really remember anything during the first week. So doctors and nurses could have told me things but I wouldn’t remember… and this is when pharmacy can help because you don’t need to go to a doctor to ask a question when you have settled down at home.”* (BW 2)
	*“It would be fantastic if you could go to one place for all the information rather than having to go here and there, you know, one place for the medical information, and another place for the herbal information. It will be like a one-stop for busy mums to get all the information they needed.”* (BW 5)
Client-pharmacist relationship	*“…because you get to know your little pharmacy, like I mostly go to one pharmacy down in XXXXX and they know me now. When I go in, they ask how is the baby going… so if they have the information, that would be much easier to just talk to them.”* (BW 5)
	*“…they [breastfeeding women] are used to the place [community pharmacy].”* (BW 8)
Staff knowledge and credibility	*“We or the public would generally trust community pharmacists as good sources of information.”* (BW 1)
	*“I suppose pharmacists are very trusted in the community to a lot of people. So if someone can go to a pharmacy, and they say we do recommend you can use these herbs to increase supply, people will be more incline to believe and try it. Whereas if it is just from word of mouth, or if you see something on TV or hear about it, there’s not actually any credibility behind the claim perhaps.”* (BW 7)
	*“It would be so much easier for new mums to just walk in [to a community pharmacy] and get some reliable answers to their queries.”* (BW 12)
Cost factors	*“It’ll be great to have someone with medical knowledge, not having to go to the doctor and spend eighty-dollars just for a question and not having to rely on the internet for basic questions to make sure I get the right information.”* (BW 2)

#### 3.1.1. Convenience and Accessibility

Participants highlighted the convenience and accessibility of community pharmacy as a facilitator to expand the role of community pharmacists in supporting breastfeeding, whilst at the same time promoting safe and effective use of medicines. Participants perceived community pharmacy as an easily accessible source of information and supply of a wide range of products.

In the context of convenience and accessibility, locality emerged as an underlying theme. Community pharmacies are spread out in the Perth metropolitan area and were perceived as “everywhere” and “local” by participants.

“My local chemist is very near my house, it is literally just behind us, I can walk there. I think that is the case for most people as there are so many pharmacies around. You don’t have to make [an] appointment, and you can ask to speak to a pharmacist.”

(BW 18)

Participants felt at ease and comfortable to discuss issues relating to breastfeeding with their local trusted community pharmacists.

“…it is just another avenue that new mothers can use. As a new mum, we are so confused and so bombarded with information that we do look for recommendations and if I knew of one that was close to me that is easy for me to get to, I think I would use them the same as how I would use my child health nurses to answer my questions… The fact that they are near and local, they are definitely very easy to access.”

(BW 13)

In addition to locality, availability was recognised as a facilitator by some participants. The opening hours and the “no appointments required” common practice of community pharmacies were valued, especially for those who labelled themselves as “busy mums”.

“I know I can go anytime, they are open quite late, and you can get the support and advice when needed because often when you are breastfeeding with the young child it is very difficult to get a doctor’s appointment or another appointment. So it’s not easy having that accessibility.”

(BW 9)

Participants labelled community pharmacy as a one-stop destination involved in many aspects of their health, from a source of information, to a source of supply and to monitoring of medical conditions in the community or an “alternative to doctors”. Some had utilised community pharmacies as a source of breastfeeding-related information, source of herbal galactagogue supply and source of advice regarding breastfeeding performance and infants’ health.

“...it is convenient because you can just buy the products or whatever they recommend at the same place, you don’t have to go and see someone, and then drive to another shop to buy the products. It’s just easier to go to one place, especially when you know you can get most of the things from one place when you are so busy with baby and other stuff.”

(BW 14)

Participants experienced community pharmacy as a provider of a vast range of health-related products and facilities. Pharmacists were expected to play the role of health and medicine-related information provider and product supplier. There was an expectation that community pharmacists should set aside their personal opinions, follow ethical obligations and have an adequate knowledge of all products available at the pharmacy.

“I would ask more questions at the place where I get my products. For example, if I go to the chemist and get my fenugreek, I would ask the pharmacist or the staff questions about the product, because I would assume they would know best because they have it in their store. I would trust the information because they are trained in that area and from my past experience, they have always been quite helpful.”

(BW 12)

#### 3.1.2. Client-Pharmacist Relationship

Participants who managed to build a trusting relationship with their local community pharmacists were more likely to perceive community pharmacy as a valuable resource.

“I just notice that my local chemist is very good with all the over-the-counter medicines, asking about what I am taking and you know, any contraindications with other medicines and things…”

(BW 10)

#### 3.1.3. Staff Knowledge and Credibility

Information attained from pharmacists was viewed as trust-worthy and credible. As some participants were concerned about the reliability of information obtained from non-reputable sources such as the internet, participants appreciated the value of information given by pharmacists.

“I think getting information or a recommendation from a pharmacist would be more reputable than your own internet search, because you don’t know how reliable that website is, it may be leading you down the wrong path, so I think a pharmacist might have the advantage of that part, plus they know what they are talking about, because they are trained in that field.”

(BW 13)

#### 3.1.4. Cost Factors

Cost factors were quoted as a reason for the role of community pharmacists to be expanded. Visits to a doctor or other health professional(s) were seen as costly to some participants, while many believed that similar information could be obtained from a pharmacist without a charge.

“…it would be less expensive this way, knowing that starting a family would cost some money, pharmacists are there all the time, you can just ask and get some answers you trust.”

(BW 16)

### 3.2. Barriers to an Increased Role of Community Pharmacists

A number of potential barriers were identified, which included the lack of advertisement, publicity and promotions, inconsistent approach, breastfeeding-related inexperience and low awareness, pharmacists’ pre-conceived perception towards herbal medicines, overlap of role with other health professionals, and privacy issues, as summarised in Table 3 with selected participant quotes.

Although many community pharmacies may be involved in expanding their services to the public, the lack of advertisement, publicity and promotions were identified by participants as a barrier. Participants commented that many women were unaware of the professional services currently available in community pharmacies, for example infant weigh-in services and appointments with lactation consultants. As the conventional scope of a community pharmacy was predominantly dispensing of prescription medicines and the supply of medicinal products, in the absence of adequate publicity, breastfeeding women were not aware of and utilising these other services. The lack of a consistent approach along with some enquiries handled by pharmacy assistants was seen as a hindrance to building a trusting relationship with the pharmacist. A lack of some pharmacists’ personal breastfeeding-related experience, knowledge and awareness was also identified as being a deterrent for breastfeeding women seeking and accepting advice.

Some participants appeared to believe that pharmacists may have pre-conceived negative perceptions towards herbal medicines. Participants who believed that there was a limited role for pharmacists to be involved in herbal remedies perceived pharmacists as “over-cautious” and fearful to recommend herbal medicines with little or no scientific evidence to support their efficacy and safety during breastfeeding, which was seen by regular users of herbal remedies as a lack of willingness to supply and inadequate knowledge in the area of CMs.

Some further expressed their concerns with regards to privacy when discussing breastfeeding-related issues in the pharmacy. The layout of some pharmacies was perceived as not facilitating privacy. Despite the availability of a vast range of products and brands across pharmacies, some participants expressed their frustrations at the lack of availability of herbal galactagogues. Many participants still believed that it would be favourable if community pharmacists were better educated in the area of herbal medicines and breastfeeding.

**Table 3:** Barriers to an increased role of community pharmacists.

Subthemes	Supporting Quotes
Lack of publicity and public awareness	*“It needs to be more advertised, or maybe mothers are told while at the hospital stay. A lot of women actually don’t know about the services available.”* (BW 2)
Inconsistent approach	*“Depends if I am shopping with the kids… if I am, they will ask [if I am breastfeeding], if not, they won’t. Most never ask me if I don’t have kids with me. Just the assumption that someone is not with a child, doesn’t mean they are not breastfeeding. You look okay, not messy, just doing shopping in nice clothes, they obviously made the assumption that you are not breastfeeding. Without kids, very rarely they ask.”* (BW 3)
Breastfeeding-related inexperience and low awareness	*“I am not sure how much they know or whether they have experience with breastfeeding and all…”* (BW 18)
	*“…if community pharmacists are better educated and have better awareness in this area or focus a lot more about safety and efficacy of medicines including herbal options during breastfeeding, that will be helpful.”* (BW 3)
Pharmacists’ pre-conceived perception towards herbal medicines	*“I think if all health professionals, not just community pharmacists, if they can be more aware of and be more open to discussion about the use of alternative therapies during breastfeeding, this would definitely help breastfeeding women. I am saying this not because I am being an advocate, it’s about giving women the choice and option that they are comfortable with…”* (BW 18)
Overlap of role with other health professionals	*“[If] it was about herbal stuff, maybe the naturopaths and friends who are naturopaths… but if it was to do with Motilium^®^ or medical or drugs, I will be talking to a lactation consultant, a GP or district nurse or pharmacists.”* (BW 4)
Privacy issues and pharmacy layout	*“…there should be a breastfeeding section…”* (BW 6)

## 4. Discussion 

Breastfeeding women who used herbal medicines including galactagogues in this qualitative study regarded pharmacists as trusted health professionals, experts in medicines, and the first port of call for questions relating to the use of medicines during breastfeeding. They also confirmed that community pharmacies were used as a source of herbal medicines information and advice on their use. Furthermore, these women expected pharmacists to have a basic knowledge of breastfeeding and the various issues related to breastfeeding.

Although pharmacists were not reported to be actively recommending the use of herbal galactagogues during breastfeeding, as one of the major suppliers of herbal medicines, the women expected pharmacists to play a substantial role in providing advice and recommendations regarding their use. This finding was in accordance with previous studies which explored consumers’ views on CMs in general and their expectations of pharmacists [19,27]. Some participants expressed a desire for pharmacists to be more “open” to discuss various issues related to breastfeeding and to offer more information on CMs during breastfeeding. Others believed that pharmacists may have a pre-conceived negative perception towards herbal medicines, and expressed a need for pharmacists to consider their use during breastfeeding. Some viewed the supply of herbal medicines and breastfeeding advice as outside the scope of pharmacy practice, causing a barrier to obtaining pharmacists’ advice. On the other hand, some participants believed pharmacists to be knowledgeable when CMs were sold in the pharmacies.

Despite the widespread use of herbal medicines, very little is currently known about their effectiveness and safety, particularly in breastfeeding [1,10,28]. This in itself is of concern considering the chemical complexities of herbal medicines and the paucity of scientific evidence to support their clinical efficacy and safety in breastfeeding. Additional risk arises for those using multiple herbal medicines. Thus one of the challenges to providing advice to consumers regarding the use of herbal medicines is the lack of quality information [29,30]. It could also be argued that poor access to the available resources or the lack of credible resources could contribute to women’s perceptions of pharmacists not having sufficient information. A need was therefore identified to address the information needs and that the available (or the lack of) information about the effectiveness and safety of these medicines needs to be disseminated to breastfeeding women in an effective manner. While the knowledge and credibility of both pharmacists and pharmacy staff were cited in this study as facilitators of the use of community pharmacists in a more active role in supporting breastfeeding, the context of discussion was often more focused on pharmacists’ knowledge on general health conditions and the use of conventional medicines and less on the use of herbal galactagogues.

From the perspectives of breastfeeding women using herbal galactagogues, the findings of this study have enhanced our understanding of the current and potential roles of pharmacists. Women in the study regarded the convenience and accessibility of community pharmacies as facilitators to obtain advice in a timely manner. This aspect, in contrast to the appointment-based services provided by most other health professionals, enhanced the role of community pharmacies in providing healthcare services to breastfeeding women. Women perceived pharmacies as a convenient one-stop health destination, to obtain health and medicine-related advice, purchase products, and receive professional services, as well as an alternative to their local general practitioners. This finding demonstrated a switch from the traditional “shopkeeper” image of pharmacists to the “healthcare provider” image [31,32]. Besides contributing to the expansion of pharmacists’ role in the community, this reduces the burden on the healthcare system and potentially frees up general practitioners for other consultations or reduces appointment waiting times.

As expected, this study has limitations. The method and process of recruitment would have resulted in selection bias. Participants were self-selected through expression of interest and hence their views may not be representative of all breastfeeding women who are regular users of herbal medicines. However, the recruitment method used was deemed most appropriate to identify or contact potential participants in this study. The fact that the interviewer (TFS) is a pharmacist may have influenced the interviews and affected the analysis. In an attempt to counterbalance the potential bias, TFS had regular meetings throughout the interview period with the third and last authors, both of whom have different disciplinary backgrounds and have no affiliations with any community pharmacies. Data were also analysed and cross-checked with the other authors to improve validity of the findings [33,34].

There is a clear need to ensure that community pharmacists are adequately trained and skilled in advising women about herbal galactagogues and their other breastfeeding enquiries. To address this issue, further studies are warranted to explore pharmacists’ knowledge of and perspectives regarding the lack of high quality evidence in this area. In addition, pharmacists need to be clear about their scope of practice and skills and be able to identify the need to refer breastfeeding women to other health professionals, for example child health nurses or lactation consultants for further breastfeeding-related advice when necessary. This would also help foster inter-professional collaboration and relationships. Finally, as indicated by the authors of the most recent review in this area, high quality studies are needed to ascertain the efficacy and safety of herbal galactagogues [12].

## 5. Conclusions 

This study reinforced the role of community pharmacists in supplying herbal galactagogues and provided insight into the reasons why women using these products do, and do not seek advice of these health professionals. There are many reasons for community pharmacists being a convenient source of information for breastfeeding women, however in relation to herbal galactagogues there is a need for both the women and the pharmacists to be aware of the current lack of high quality evidence for their efficacy and safety. Well-designed studies with adequate numbers of participants are required in order to establish these parameters.
